# [18F]flutemetamol uptake in the colon of a memory clinic population and its association with brain amyloidosis and the gut microbiota profile: an exploratory study

**DOI:** 10.1007/s00259-025-07299-8

**Published:** 2025-05-02

**Authors:** Giulia Quattrini, Elena Gatti, Débora Elisa Peretti, Marco Aiello, Claire Chevalier, Aurelien Lathuiliere, Rahel Park, Michela Pievani, Marco Salvatore, Max Scheffler, Annamaria Cattaneo, Giovanni B. Frisoni, Valentina Garibotto, Moira Marizzoni

**Affiliations:** 1 Laboratory of Alzheimer’s Neuroimaging and Epidemiology (LANE), IRCCS Istituto Centro San Giovanni di Dio Fatebenefratelli, Brescia, 25125 Italy; 2 Laboratory of Neuroimaging and Innovative Molecular Tracers, Geneva University Neurocenter and Faculty of Medicine, University of Geneva, Geneva, Switzerland; 3 IRCCS SYNLAB SDN, Naples, 80143 Italy; 4 Geneva Memory Center, Geneva University Hospitals, Geneva, Switzerland; 5 Laboratory of Neuroimaging of Aging (LANVIE), University of Geneva, Geneva, Switzerland; 6 Division of Radiology, Geneva University Hospitals, Geneva, Switzerland; 7 Biological Psychiatric Unit, IRCCS Istituto Centro San Giovanni di Dio Fatebenefratelli, via Pilastroni 4, Brescia, 25125 Italy; 8 Department of Pharmacological and Biomolecular Sciences, University of Milan, Milan, Italy; 9 Division of Nuclear Medicine and Molecular Imaging, Geneva University Hospitals, Geneva, Switzerland; 10 Center for Biomedical Imaging, Geneva, Switzerland

**Keywords:** Alzheimer’s disease, Amyloid PET, Colon, Gut microbiota

## Abstract

**Purpose:**

Some Alzheimer’s disease (AD) patients report gastro-intestinal symptoms and present alterations in the gut microbiota (GM) composition. Elevated colonic amyloid immunoreactivity has been shown in patients and animal models. We evaluated the colonic uptake of the amyloid positron emission tomography (PET) imaging agent [18F]flutemetamol (FMM) in a memory clinic population and investigated its association with brain amyloidosis and GM composition.

**Methods:**

Forty-five participants underwent (i) abdominal and cerebral FMM PET, acquired at 40 (early phase) and 120 min (late phase) after tracer injection, (ii) abdominal computed tomography, and (iii) cerebral T1-weighted MRI. Colonic standardized uptake value ratio (SUVr) was determined through manual tracing and automatic segmentation (TotalSegmentator), using the aortic blood signal as a reference region. Fecal GM composition was assessed using 16 S rRNA sequencing. Amyloid positive (A+) and negative (A-) participants, based on cortical FMM quantification (PetSurfer), were compared in terms of SUVr and GM features.

**Results:**

Increased colonic early SUVr was reported in A+ than A- (manual, *p* =.008; automated, *p* =.035). Altered GM composition was found in A + as shown by lower Pielou’s evenness (*p* =.023), lower abundance of *Eubacterium hallii group*, and higher abundance of several genera. High *UC5-1-2E3* abundance positively correlated with high colonic early SUVr (whole group: manual, *p* =.012, automated, *p* =.082; A+: manual, *p* =.074; automated, *p* =.016).

**Conclusion:**

This exploratory study showed that subjects with cerebral amyloidosis have greater colonic FMM uptake than subjects with normal cerebral amyloid load, correlating with altered GM composition. Further analysis is needed to determine if these changes denote amyloid-related changes or other phenomena.

**Supplementary Information:**

The online version contains supplementary material available at 10.1007/s00259-025-07299-8.

## Introduction

Alzheimer’s disease (AD) is characterized by central and peripheral pathophysiological alterations. The former include an extracellular accumulation of amyloid beta (Aβ), intracellular depositions of hyper-phosphorylated tau, and neurodegeneration in specific brain regions [1]. The latter involve gastro-intestinal symptoms, which also emerged as risk factors and could represent early symptoms associated with dementia [2–7].

Increasing evidence supports the involvement of the gut microbiota (GM) in promoting AD onset and progression. Preclinical studies found that GM is necessary for brain amyloid deposition [8–10]. Clinical studies showed that AD patients have a peculiar GM profile, enriched in pro-inflammatory bacteria, that correlates with cerebrospinal fluid and positron emission tomography (PET) markers of brain amyloid pathology [11–13].

Non-invasive imaging tools represent a unique opportunity to assess the involvement of peripheral organs in AD pathophysiology. PET imaging is largely applied to visualize and quantify Aβ plaques in the brain [14, 15]. Interestingly, the amyloid precursor protein is normally expressed by enteric neurons and glia [16, 17] and colonic Aβ deposition has been reported in patients with neuropathologically-confirmed AD, as well as in a proportion of older unimpaired adults [18, 19]. Although these data suggest that AD processes might occur outside the brain, to the best of our knowledge, *in-vivo* data on amyloid deposition in the colon are still inexistent.

Objectives of this exploratory study were to quantify the uptake of the [18 F]flutemetamol [FMM] amyloid tracer [15, 20, 21] in the colon of a memory clinic population and to evaluate its association with cerebral amyloid pathology and GM composition.

## Materials and methods

### Participants

Fifty-one participants were enrolled from the gMAD/COSCODE cohort at the Centre de la Mémoire of Geneva University Hospitals (https://www.hug.ch/centre-memoire) from January 2021 to May 2022. Subjects were between 55 and 80 years of age with any level of cognitive functioning (i.e. dementia, mild cognitive impairment, subjective cognitive decline, worried-well, healthy controls). Cognitive impairment was defined based on the presence of objective evidence of cognitive impairment and cognitive concern reported by the patient and/or informant (family or close friend) [22]. Exclusion criteria were the presence of autoimmune and chronic inflammatory diseases, active gastrointestinal disease, anti-inflammatory or antibiotic treatment over the past 3 months, or a history of previous cholecystectomy. The gMAD/COSCODE study was approved by the competent ethics committee (Commission cantonale d’éthique de la recherche [CCER]; reference No. CCER_2016 − 01346, CCER_2020_00403) and was conducted in accordance with the first revision of the Declaration of Helsinki from 1975.

### Imaging acquisition protocols

Neuroimaging data was based on 3T MRI (SIEMENS MAGNETOM Skyra) and PET/CT exams, acquired according to the following protocols: (i) 3D T1-weighted Magnetization Prepared RApid Gradient Echo (MPRAGE) sequence with 1 mm^3^ isotropic voxel-size, TR = 1810 ms, TE = 2.19 ms, acceleration factor = 3, flip angle = 8°; (ii) amyloid PET, 90 min after injection of 150 MBq (FMM), 4 × 5-minutes image frames.

The abdominal imaging protocol included two PET/CT exams of the whole abdominopelvic region acquired (i) during the interval between early and late acquisition phases of the brain PET/CT scan, at 40 min post tracer injection (early phase; e-FMM), and (ii) after the end of the late brain acquisition, at 120 min post tracer injection (late phase; l-FMM) (Supplementary Fig. 1). Protocols were as follows: (i) PET voxel size 1.65 × 1.65 × 1.65 mm^3^ or 4.07 × 4.07 × 2.5 mm^3^; (ii) CT, voxel size 0.64 × 0.64 × 1.40 mm^3^ or 1 × 1 × 1 mm^3^. Fasting during 4 h before the tracer injection was recommended for both cerebral and abdominal PET/CT. All images were acquired using a Siemens Biograph PET scanner (Siemens Medical Solutions, USA), reconstructed using a 3D OSEM algorithm (4 iterations, 8 subsets), a 2 mm Gaussian convolution kernel, corrected for dead time, normalization, attenuation, and sensitivity. All commercially available radiotracers were synthesized at radiopharmaceutical Good Manufacturing Practice laboratories.

### Imaging processing

Structural MPRAGE images were visually inspected according to a published rating system [23] and then pre-processed using the FreeSurfer v7.2 standard procedure (https://surfer.nmr.mgh.harvard.edu/) [24]. The quality control of outputs was conducted using Qoala-T (https://github.com/Qoala-T/QC) [25] and none was excluded. Cerebral amyloid l-PET scans were first smoothed (8 mm FWMH) using the Statistical Parametric Mapping tool, v12 (SPM12; https://www.fil.ion.ucl.ac.uk/spm/). Then, PetSurfer (https://surfer.nmr.mgh.harvard.edu/fswiki/PetSurfer) [26, 27] was used to extract the mean intensity values from each cortical label of the Desikan-Killiany atlas. The weighted mean for a composite cortical region (frontal, anterior and posterior cingulate, lateral parietal, and lateral temporal) was normalized to the whole cerebellum, to derive the cortical standardized uptake value ratio (SUVr) [28]. Participants were classified into negative (A-) and positive (A+) based on the cerebral amyloid fixation status, according to the established cut-off for FMM (SUVr > 1.03), reported as highly concordant with PET visual reading [29].

**Fig. 1:**
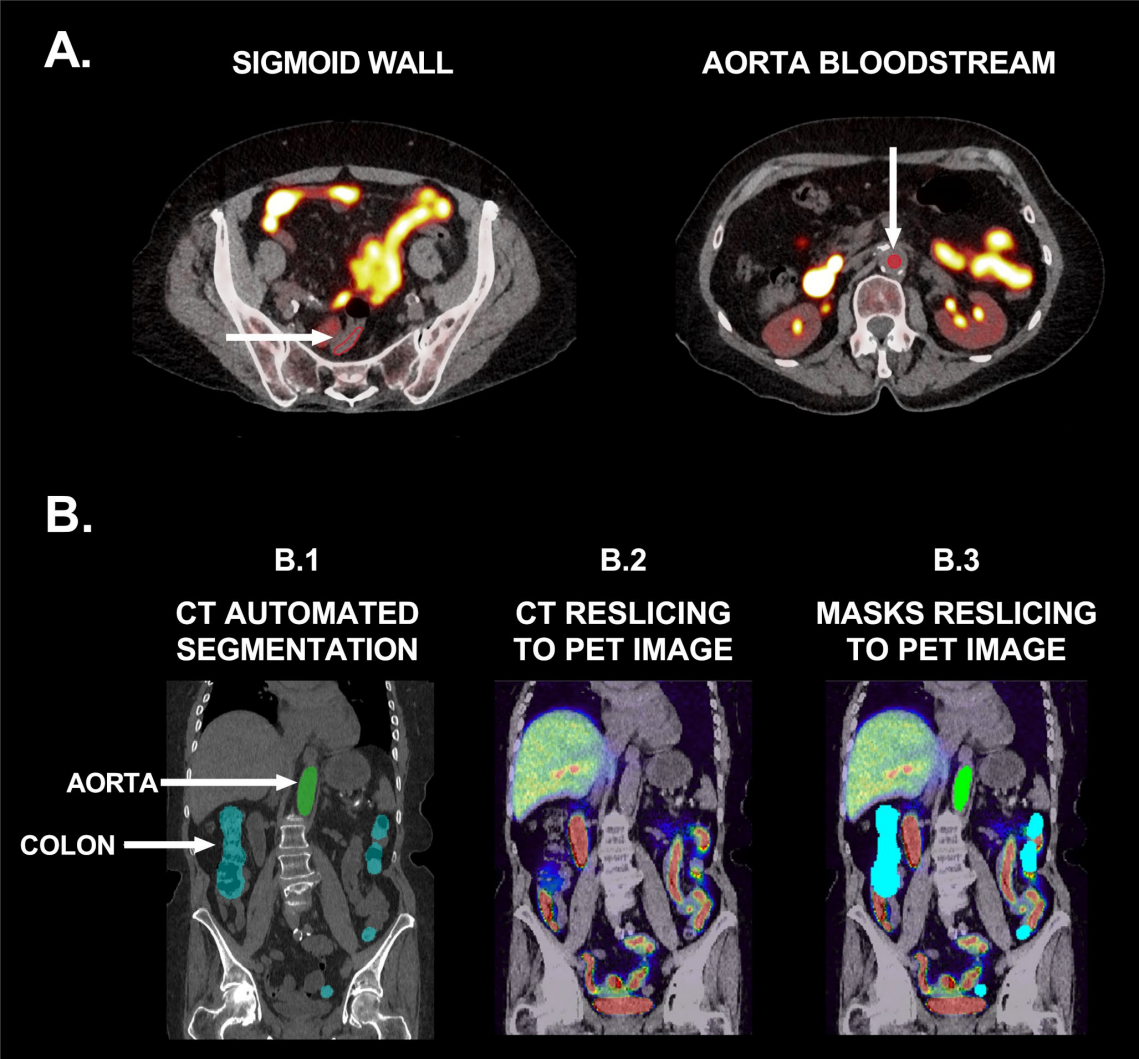
Manual and automated segmentation procedures of volumes of interest. Manual procedure (**panel A**): volumes of interest for the sigmoid wall and aorta bloodstream were manually drawn on CT. Automated procedure (**panel B**): (i) aorta (green) and colon (cyan) binary masks were automatically computed (**panel B.1**); (ii) CT images were resliced to PET space (**panel B.2**); (iii) the inverse transformation was applied to aorta and colon binary masks to match PET resolution, and the uptake values were computed (**panel B.3**)

Abdominal FMM uptake was quantified using two independent methodologies: manual tracing, and automated quantification (Fig. 1). For manual tracing, volumes of interest (i.e., aortic lumen, wall of the sigmoid colon) were manually drawn on a single PET/CT slice by a board-certified specialist in nuclear medicine (VG) using the OsiriX MD software (https://www.osirix-viewer.com) [30] as shown in Fig. 1. The aortic lumen was selected in order to avoid any potential confounding effect due to FMM binding to atherosclerotic plaques [31]. The tracing was performed drawing a circular region (diameter: 1 cm) at the level of the left kidney artery/vein. The sigmoid was drawn at the S1-S2 level on the posterior sigmoid wall as a hollow volume, to only include the signal from the tissue. The ratio between the mean and maximum FMM SUV of the bowel wall and the aortic lumen (reference) was computed to derive the organ SUVr. For the automated segmentation, early and late CT scans were automatically segmented using Total Segmentator v2 (https://github.com/wasserth/TotalSegmentator) [32], to obtain binary masks of colon and aorta (Fig. 1, panel B.1). To isolate the bloodstream signal and to exclude the binding to atherosclerotic plaques on the vessel wall [31], the aorta mask was eroded by 1 voxel. The Advanced Normalization Tools (ANTs) (http://stnava.github.io/ANTs/; https://github.com/ANTsX/ANTs) v2.3.5 [33] was used to compute the affine transformation between the PET and the fusioned CT (Fig. 1, panel B.2). Then, by applying the inverse affine transformation, the binary masks of colon and aortic bloodstream were aligned and resliced onto the PET space (Fig. 1, panel B.3). A visual inspection was conducted across all steps. One late PET acquisition was excluded due to procedure failure. The colon and aortic bloodstream masks were overlaid on the matched PET image and the underling (i) mean and (ii) mean over the 99th percentile (proxy of maximum value) FMM intensity were computed using the FMRIB Software Library (FSL) (https://fsl.fmrib.ox.ac.uk/fsl/fslwiki/) [34]. Finally, the ratio between the mean and maximum FMM uptake of colon and aortic bloodstream was computed to derive the organ SUVr.

### Fecal microbiota composition

Bacterial DNA was extracted from 50 mg of frozen stools with the MagPurix Bacterial DNA Extraction kit using an automated system (Zinexts, New Taipeh City, ROC), according to the manufacturer’s instructions. DNA was quantified using a NanoDrop ND-1000 spectrophotometer (Ozyme, Saint-Cyr-l’Ecole, France), and then stored at -20 °C for subsequent analyses. Regions V3 and V4 of the bacterial 16S sub-unit of the ribosomal RNA (16 S rRNA) gene were amplified and purified according to the 16S Metagenomic Sequencing Library Preparation protocol by Illumina (San Diego, CA, USA). The amplicons were pooled in equal concentration and sequenced with Illumina MiSeq PE300. The raw 16S rRNA gene data were processed using QIIME2 [35] (64 bit version 2024.2). Sequencing data were already demultiplexed. Forward and reverse primers, reads containing ambiguous bases or homopolymers greater than eight base pairs in length were removed. Moreover, we set a maximum number of expected errors equal to 2 and reads truncation if the quality score was less than 2. The DADA2 denoising process was applied with the default parameters [36]. Alpha diversity, indicating the richness and abundance of amplicon sequence variants within each individual, and beta diversity, showing the similarity, or difference in microbiota composition between individuals, were calculated using the q2-diversity plugin (https://github.com/qiime2/q2-diversity) after rarefaction of the feature table at the sample depth of 3887 (sample depth corresponding to the sample with the lowest read count). Alpha and beta diversity included Bray-Curtis and Jaccard indexes and Shannon and Pielou’s evenness indexes. Taxonomy was assigned to amplicon sequence variants using the q2-feature‐classifier [37] classify‐sklearn naïve Bayes taxonomy classifier against SILVA reference database (version 138) (https://www.arb-silva.de/). Absolute abundances at the genera were normalized by the total number of reads assigned in each sample. Genera with incomplete taxonomy, assigned to the Eukaryota domain or found in less than 0.1% of total samples were removed. A total of 124 ASVs were identified in the whole group after filtering.

### Statistical analysis

Statistical analyses were performed using R software package (R Foundation for statistical computing, https://www.r-project.org/; version v4.1.1) and the Rstudio GUI (http://www.rstudio.com/; version 1.3.1073). For the comparison between A+ and A- groups, descriptive statistics were performed using the Wilcoxon-Mann-Whitney rank*-*sum test or the Pearson chi-squared test for continuous and categorical variables, respectively.

Colonic SUVr values were log-transformed to normalize data distribution, when appropriate. General Linear Models (GLM) were performed with imaging or Pielou’s evenness as the dependent variable and amyloid status as the independent variable. Age was included as a covariate in all models, abdominal PET voxel size (i.e., size 1 = 1.65 × 1.65 × 1.65 mm^3^; size 2 = 4.07 × 4.07 × 2.5 mm^3^) only in those with imaging variables. Extreme outliers were identified within A- and A+ groups using the interquartile range criterion (R package *rstatix* v0.7.2) [38] and removed. For other microbiota variables, beta diversity was analyzed using permutational multivariate analysis of variance adjusted for age (PERMANOVA, R package: *vegan* v2.6-4) [39], and differential abundance of microbial genera using the Linear discriminant analysis Effect Size (LEfSe, R package: *microbial* v0.0.21) with default settings. Principal Coordinate Analysis (PCoA) was used to visualize beta diversity results. Associations of imaging and GM variables were assessed with Spearman correlation. The effect of age and PET voxel size were calculated using the Partial and Semi-Partial (Part) Correlation using the R package *ppcor* v 1.1 [40]. Significance level (*p*) was set to *p* <.050 and, for the exploratory association analyses, to *p* <.100 (two-tailed).

## Results

Table 1 summarizes the sample features of study participants according to their brain amyloid status. A+ were significantly older (*p* =.002) and, as expected for this population, were more frequently cognitively impaired (*p* <.001), and had a higher incidence of APOEε4 than A-. Groups were comparable for sex and years of education (*p* >.343).

**Table 1:** Participants’ features based on brain amyloid status. Values are reported as mean (*M*) ± standard deviation (*SD*) and range (minimum-maximum), or percentage (%). *P* denotes the statistical significance of the Wilcoxon-Mann-Whitney rank test (continuous variables) and the pearson chi-square test (categorical variables). Significant results are reported in **bold**

Demographic features	Amyloid negative M ± SD [range]	Amyloid positive M ± SD [range]	*p*
*N*	35	10	
Age, years	67 ± 8 [54–83]	76 ± 6 [64–82]	**0.002**
Sex, % females (%)	74	70	0.787
Education, years	14 ± 3[9–24]	13 ± 3 [7–16]	0.343
MMSE, score	28 ± 1 [26–30]	26 ± 2 [23–28]	**< 0.001**
Cognitively impaired (%)	23	90	**< 0.001**
APOE ε4 carriers (%)	11	50	**0.001**
FMM cortical SUVr	0.8 ± 0.1 [0.7-1.0]	1.3 ± 0.2[1.2–1.8]	**< 0.001**

Abbreviations: *N*, number of individuals; MMSE, Mini-Mental State Examination; APOE, Apolipoprotein E gene; FMM, [18F]Flutemetamol; SUVr, standardized uptake value ratio.

### Cerebral Aβ pathology was associated with higher early FMM uptake in the gut

**Fig. 2:**
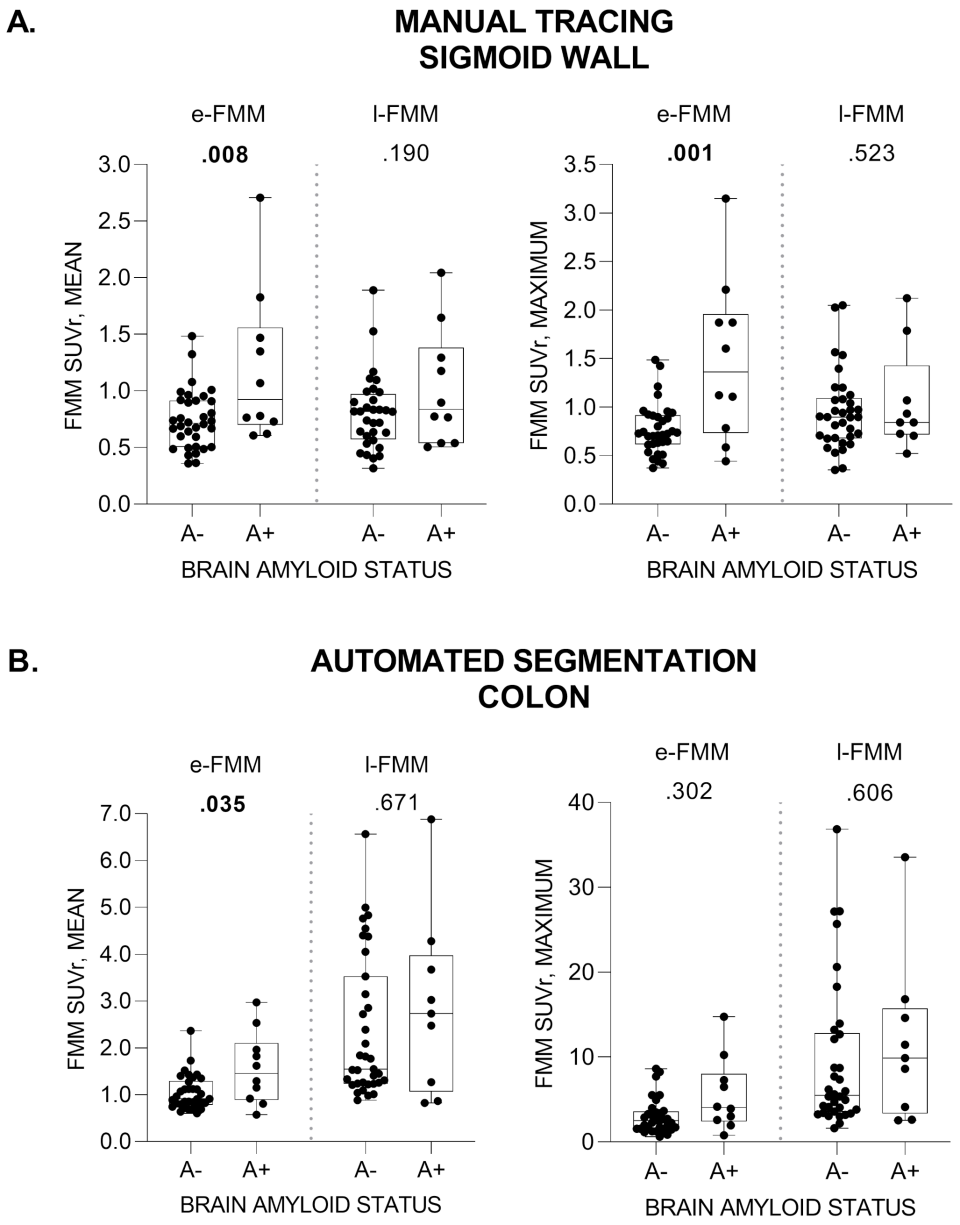
Amyloid pathology status in the brain (negative [A-] / positive [A+]) on [18 F]Flutemetamol (FMM) mean and maximum SUVr of sigmoid wall (manual tracing; **panel A**) and colon (automated segmentation; **panel B**). Early and late PET phases (e-FMM and l-FMM, respectively) were tested. Each dot represents a subject, whiskers denote the values’ range, the horizontal line within boxes is the group median. Significant results are reported in bold.

When considering manual tracing, higher mean (*p* =.008) and maximum (*p* =.001) SUVr values were reported in the sigmoid colon of A+ than of A- subjects for the early phase (Fig. 2, panel A), but not for the late phase (*p* >.190). The higher SUVr mean uptake in A+ compared to A- in the early phase was confirmed when regarding the automated segmentation (*p* =.035) (Fig. 2, panel B). No effect was reported for maximum values (*p* =.302) and for late SUVr (*p* >.606). These changes were irrespective of APOE ε4 carrier status, which only affected the mean SUVr uptake in the sigmoid colon for the late phase (*p* =.029, Supplementary Fig. 2).

### Cerebral Aβ pathology was associated with altered GM composition

**Fig. 3:**
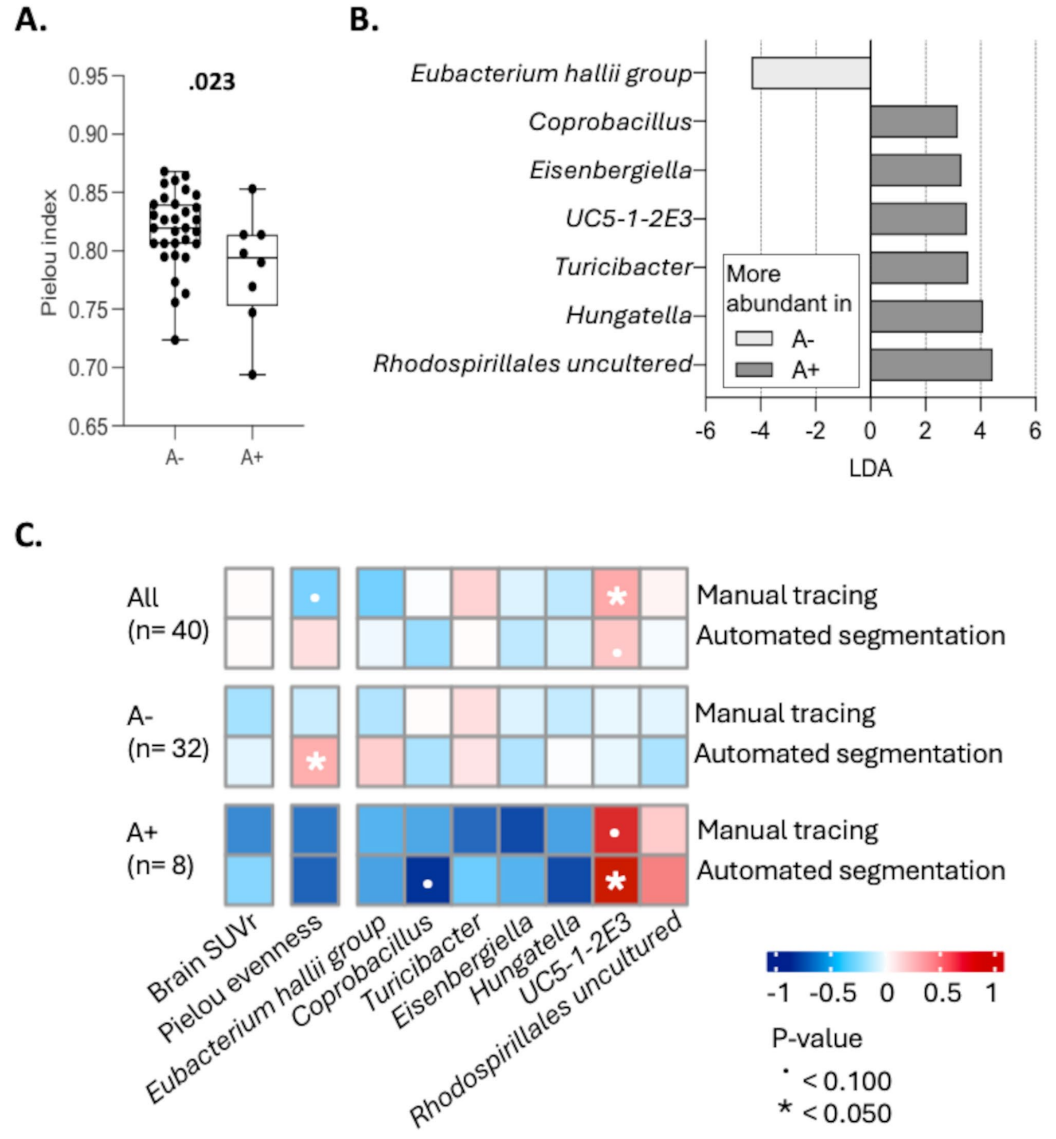
Amyloid pathology status in the brain (negative [A-] / positive [A+]) and alpha diversity measured by Pielou’s index (**panel A**) and on fecal microbiota composition at the genus level (**panel B**, linear discriminant analysis (LDA) effect size (LEfSe) algorithm, LDA > 3). Association matrix (**panel C**) between [18F]flutemetamol mean SUVr in the gut based on manual tracing (sigmoid) and automated segmentation (colon) with MMSE, cerebral mean SUVr and fecal microbiota features.

Analysis of gut microbiota composition by bacterial 16S rRNA gene sequencing revealed an altered microbiota balance in A+ compared with A- subjects, as suggested by the reduction of Pielou’s evenness (Fig. 3, panel A). Pielou’s evenness represents the distribution of abundance across the species in a community and ranges from 0 (no evenness, some species numerically dominate over others) to 1 (complete evenness, organisms are evenly distributed among species). No differences were reported for Shannon and beta diversity diversity indexes (Supplementary Fig. 3). At the genus level, A+ had on average a significant reduction in the relative abundance of *Eubacterium halii group* as well as an increase in *Coprobacillus*, *Eisenbergiella*, *UC5-1-2E3*, *Turicibacter*, *Hungetella*, and *Rhodospirillales uncultered*, when compared to A- (Fig. 3, panel B). There was no variability in gut microbiota richness and structure between APOE ε4 carrier and non-carriers (Supplementary Fig. 4, panels A-B). Genus comparison revealed that APOE ε4 carriers showed a lower abundance of *Eubacterium halii group* and *Dorea*, as well as a higher abundance of *Eubacterium xylanophilum group*, *Alistipes*, and *Eubacterium eligenes group* (Supplementary Figure, panel C). Among those, *Eubacterium halii group* was the only genus found to be different even between A+ and A- groups.

### Colon FMM uptake was associated with altered GM composition

Next, to better understand whether microbiota alterations were associated with PET changes, we correlated Pielou’s evenness and the genera found differently abundant in A+ and A- with early mean SUVr in the colon (Fig. 3, panel C). Microbiota balance (higher Pielou’s evenness values) weakly correlated with low early SUVr computed with manual tracing (*rho*=-0.29, *p* =.081). Furthermore, high *UC5-1-2E3* abundance correlated with high early SUVr, detected with both manual (*rho* = 0.40, *p* =.012) and automated (*rho* = 0.29, *p* =.082) methods. When A- and A+ were analyzed separately, the strongest associations were observed in A+, where the directions of the associations were in full agreement between the 2 methods. In detail, low abundance of *Coprobacillus* (automated, *rho*=-0.77, *p* =.076) and high abundance of *UC5-1-2E3* (manual, *rho* = 0.77, *p* =.074; automated, *rho* = 0.90, *p* =.016) were correlated with high early SUVr.

## Discussion

This study aimed at exploring the hypothesis that the colon is implicated in the pathophysiology of AD, as proposed for Parkinson’s disease (PD) [41, 42], another neurodegenerative condition associated with aging and sharing neuropathological features and molecular comorbidities with AD [43–45]. Here, we investigated the colonic uptake of the amyloid imaging agent FMM in a memory clinic population to evaluate its association with cerebral amyloidosis and stool GM composition.

The exploratory results showed that colon FMM PET enables the detection of uptake differences between A+ and A- subjects, as defined based on cerebral amyloid quantification. Furthermore, manual tracing and automated segmentation agreed in identifying associations between the colonic FMM uptake and the abundance of some bacteria in the stools. These two methodologies pointed different districts of the same organ: while manual tracing focused on colonic wall, the automated also included the colonic content. The presence of colonic content probably impacted the SUVr quantification in the automated procedure, which might lowered the statistical power to detect group differences.

Our results also showed that early but not late PET acquisitions were able to identify colonic SUVr differences between subjects with pathological brain amyloid deposits, when compared with those without deposits. The late phase is the time window in which the FMM tracer binds to target deposits in the brain [21, 46]. As the blood-gut barrier has a less stringent permeability than the blood-brain barrier [47, 48], we speculated that the tracer would be able to reach its target earlier in the colon than in the brain. However, further data such as dynamic PET acquisitions are needed to verify this hypothesis. To the best of our knowledge, this is the first study that used an amyloid PET tracer to evaluate the involvement of peripheral organs, such as the colon, in AD pathophysiology in humans. Previous studies on amyloid PET imaging outside the brain were focused on describing the tracer’s biodistribution, lacking to define the colon as a region of interest [49], or to compare with a control group [50]. The colon is closely interconnected with the brain through the vagus nerve [51] and recent clinical and pathological findings [52–56] led to the idea of a gut-brain route of alpha-synuclein pathology in PD. Similarly, AD animal studies suggested that Aβ deposits could propagate along the gut-brain axis following a prion-like mechanism [57, 58]. Colonic amyloid deposition was detected in different AD mouse models [16, 59, 60], even before the full development of brain pathology [61]. Vagal and cerebral amyloidosis were observed in mice after the injection of Aβ_1−42_ into the peritoneal cavity [62], stomach, and colon [63]. Vagotomy resulted in decreased amyloid propagation [64]. Taken together, the evidence suggest that PD and AD might share common causal mechanisms, such as the alteration of the microbiota-gut-brain axis.

The present findings confirmed that the gut microbiota is an important susceptibility factor in AD and are in line with a growing number of studies that show alterations in the GM composition in human and rodent models [10, 11, 13, 65, 66]. Specifically, A+ were found to be characterized by a reduction of the anti-inflammatory genera *Eubacterium hallii group*, also involved in the production of short-chain fatty acids, which play an important role in intestinal homeostasis [67, 68]. Conversely, A+ showed an increased abundance of the genera *Eisenbergiella*, *Hungatella*, *Coprobacillus*, *Turicibacter*, and *UC5-1-2E3. Eisenbergiella* increased with the severity of cognitive impairment in patients with dementia [69] and the increased abundance of *Hungatella* in A+ participants in our study is in line with alterations found in PD patients [69, 70]. High abundance of *Coprobacillus* and *Turicibacter* in colonic tissues correlated with metabolic alterations (i.e., deficiency in unsaturated fatty acids and choline, overabundance of ketone bodies, lactate, amino acids, trimethylamine [TMA] and trimethylamine N-oxide [TMAO]) as well as cerebral hypometabolism in an AD mouse model [71]. However, other studies suggest a protective role for *Coprobacillus* as a butyrate-producing genus [72, 73] with a role in maintaining intestinal stability and conferring resistance to *Clostridium difficile* colonization [74]. This would be in line with the mild association of *Coprobacillus* with low SUVr in the A + group but, given the limited number of A + and the absence of literature refuting or confirming these preliminary results, extreme caution is needed in the interpretation. *UC5-1-2E3* has been recently proposed as novel microbial biomarker to predict metabolic traits associated with prediabetes [75], a condition that increased the risk to develop dementia [76]. These results support the notion that gut dysbiosis and changes in bacterial metabolite production might represent one of the mechanisms underlying some of the gut signs and symptoms observed in AD [2–4]. Of interest, the abundance of *UC5-1-2E3* positively correlated with the SUVr uptake in the colon in the whole cohort as well as in the A+ group. Although this study did not demonstrate that colonic FMM uptake is due to the presence of amyloid aggregates, it suggested a possible association between this phenomenon and gut bacteria. Bacterial amyloids display structural similarity with the human amyloids, facilitated protein aggregation in-vitro [77] as well as in PD rat models and C. elegans [78], and might argument inflammatory responses to endogenous amyloids [79]. Moreover, human amyloids are physiologically produced by the immune system in response to microbial invasion [80]. The disruption of the mucus layer and the intestinal-blood barrier found in several central disorders, including AD [81], could facilitate cell immune activation by intestinal bacteria that move from the lumen to the epithelium thus promoting local amyloid production [82]. Consistent with this hypothesis, an animal model knock-out of a gene important for mucosal protection resulted in an increased abundance of *UC5-1-2E3* together with a reduced goblet cell count and mucin expression [83]. In addition, a genetic polymorphism within the exon of Mucin 6 has been recently found to be associated with late-onset AD and the severity of phosphorylated tau pathology in neocortex [84].

A limitation of this pilot study is the small sample size, especially of the A+ group, that limited the statistical power of the exploratory association analyses where, despite the high correlation coefficients, few were significant. Future studies including larger datasets could confirm - or not - the association between colonic FMM uptake and gut bacteria’s prevalence, and evaluate their possible involvement at earlier stage of the disease. A second limitation lies in the lack of a validation of our imaging protocol for abdominal acquisitions with gold standard assessment of amyloid uptake in the colonic wall, which prevent us to draw firm conclusions on the association of FMM distribution in the colon to amyloid pathology. Giving the exploratory nature of this study, the acquisition of the abdominal PET scan was performed within the scanning time window already used for brain imaging. This guaranteed that the signal coming from the intestinal content remained low, in line with the known FMM biodistribution [85]. Moreover, since the PET protocol gave priority to brain imaging, abdominal perfusion imaging over the first minutes was not possible. Future studies would benefit from an optimization of the acquisition protocol for abdominal PET imaging. Finally, the manual tracing specifically targeted the sigmoid wall and the automated segmentation computed the whole colon. We cannot exclude a potential confounding effect of the intestinal content in the FMM uptake quantification from the automated procedure, and the usage of two different voxel sizes prevented us to consistently extract the colon wall across participants. On the other hand, we applied an automatic procedure that was as simple and consistent as possible to make it easily applicable to any abdominal PET scan, regardless of voxel size or used tracer. Finally, it should be noted that A+ and A- participants were not balanced by APOE ε4 carrier status and age, two factors that may influence both imaging features and the gut microbiota [86–89]. Although all the models were corrected for age and no biological APOE ε4- associated differences emerged in the present analyses (Supplementary Fig. 2 and Supplementary Fig. 4), future research investigating the role of these two factors in abdominal imaging and gut microbiota diversity in the context of AD is necessary.

## Conclusion

This exploratory study provided *in-vivo* preliminary evidence of a different FMM uptake in the colon of A+ and A- subjects from a memory clinic population, which correlated with GM composition. Even though more investigation is required to determine the nature of these differences, and whether they denote amyloid-related changes or other phenomena, these results provide a basis for future studies on the gut-brain axis in relation to AD. Understanding the influence of the gut microbiota on gut and brain health could lead to the development of novel therapeutic and preventive agents.

## Electronic supplementary material

Below is the link to the electronic supplementary material.

Supplementary Material 1

## Data Availability

The datasets generated during the current study are available on request.

## References

[1] Scheltens P, Blennow K, Breteler MMB, de Strooper B, Frisoni GB, Salloway S, et al. Alzheimer’s disease. Lancet. 2016;388:505–17. 10.1016/S0140-6736(15)01124-1 26921134 10.1016/S0140-6736(15)01124-1

[2] Hernández-Ruiz V, Roubaud-Baudron C, Von Campe H, Retuerto N, Mégraud F, Helmer C, et al. Association between Helicobacter pylori infection and incident risk of dementia: the AMI cohort. J Am Geriatr Soc. 2024;72:1191–8. 10.1111/jgs.18748 38258504 10.1111/jgs.18748

[3] Huang J, Su B, Karhunen V, Gill D, Zuber V, Ahola-Olli A, et al. Inflammatory diseases, inflammatory biomarkers, and alzheimer disease: an observational analysis and Mendelian randomization. Neurology. 2023;100:e568–81. 10.1212/WNL.000000000020148 36384659 10.1212/WNL.0000000000201489 PMC9946179

[4] Adewuyi EO, O’Brien EK, Porter T, Laws SM. Relationship of cognition and Alzheimer’s disease with Gastrointestinal tract disorders: A Large-Scale genetic overlap and Mendelian randomisation analysis. Int J Mol Sci. 2022;23:16199. 10.3390/ijms232416199 36555837 10.3390/ijms232416199 PMC9784325

[5] Jung JH, Kim G, Byun MS, Lee JH, Yi D, Park H, et al. Gut Microbiome alterations in preclinical Alzheimer’s disease. PLoS ONE. 2022;17:e0278276. 10.1371/journal.pone.0278276 36445883 10.1371/journal.pone.0278276 PMC9707757

[6] Kountouras J, Tsolaki M, Gavalas E, Boziki M, Zavos C, Karatzoglou P, et al. Relationship between Helicobacter pylori infection and alzheimer disease. Neurology. 2006;66:938–40. 10.1212/01.wnl.0000203644.68059.5 16567719 10.1212/01.wnl.0000203644.68059.5f

[7] Chen CH, Lin CL, Kao CH. Irritable bowel syndrome is associated with an increased risk of dementia: A nationwide population-based study. PLoS ONE. 2016;11:e0144589. 10.1371/journal.pone.0144589 26731277 10.1371/journal.pone.0144589 PMC4701489

[8] Kim M-S, Kim Y, Choi H, Kim W, Park S, Lee D, et al. Transfer of a healthy microbiota reduces amyloid and Tau pathology in an Alzheimer’s disease animal model. Gut. 2020;69:283–94. 10.1136/gutjnl-2018-317431 31471351 10.1136/gutjnl-2018-317431

[9] Sun J, Xu J, Ling Y, Wang F, Gong T, Yang C, et al. Fecal microbiota transplantation alleviated Alzheimer’s disease-like pathogenesis in APP/PS1 Transgenic mice. Transl Psychiatry. 2019;9:189. 10.1038/s41398-019-0525-3 31383855 10.1038/s41398-019-0525-3 PMC6683152

[10] Harach T, Marungruang N, Duthilleul N, Cheatham V, Coy KDM, Frisoni G, et al. Reduction of Abeta amyloid pathology in APPPS1 Transgenic mice in the absence of gut microbiota. Sci Rep. 2017;7:41802. 10.1038/srep41802 28176819 10.1038/srep41802 PMC5297247

[11] Cattaneo A, Cattane N, Galluzzi S, Provasi S, Lopizzo N, Festari C, et al. Association of brain amyloidosis with pro-inflammatory gut bacterial taxa and peripheral inflammation markers in cognitively impaired elderly. Neurobiol Aging. 2017;49:60–8. 10.1016/j.neurobiolaging.2016.08.019 27776263 10.1016/j.neurobiolaging.2016.08.019

[12] Ferreiro AL, Choi J, Ryou J, Newcomer EP, Thompson R, Bollinger RM, et al. Gut Microbiome composition May be an indicator of preclinical Alzheimer’s disease. Sci Transl Med. 2023;15:eabo2984. 10.1126/scitranslmed.abo2984 37315112 10.1126/scitranslmed.abo2984 PMC10680783

[13] Vogt NM, Kerby RL, Dill-McFarland KA, Harding SJ, Merluzzi AP, Johnson SC, et al. Gut Microbiome alterations in Alzheimer’s disease. Sci Rep. 2017;7:13537. 10.1038/s41598-017-13601-y 29051531 10.1038/s41598-017-13601-y PMC5648830

[14] D’Amico F, Sofia L, Bauckneht M, Morbelli S, Amyloid PET, Imaging. Standard procedures and semiquantification. Methods Mol Biol. 2024;2785:165–75. 10.1007/978-1-0716-3774-6_11 38427194 10.1007/978-1-0716-3774-6_11

[15] Meyer PF, McSweeney M, Gonneaud J, Villeneuve S. AD molecular: PET amyloid imaging across the Alzheimer’s disease spectrum: from disease mechanisms to prevention. Prog Mol Biol Transl Sci. 2019;165:63–106. 10.1016/bs.pmbts.2019.05.001 10.1016/bs.pmbts.2019.05.001 31481172

[16] Cabal A, Alonso-Cortina V, Gonzalez-Vazquez LO, Naves FJ, Del Valle ME, Vega JA. β-Amyloid precursor protein (βAPP) in human gut with special reference to the enteric nervous system. Brain Res Bull. 1995;38:417–23. 10.1016/0361-9230(95)02006-D 8665264 10.1016/0361-9230(95)02006-d

[17] Arai H, Lee VM, Messinger ML, Greenberg BD, Lowery DE, Trojanowski JQ. Expression patterns of β-amyloid precursor protein (β‐APP) in neural and nonneural human tissues from Alzheimer’s disease and control subjects. Annals Neurology: Official J Am Neurol Association Child Neurol Soc. 1991;30:686–93. 10.1002/ana.410300509 10.1002/ana.410300509 1763893

[18] Joachim CL, Mori H, Selkoe DJ. Amyloid β-protein deposition in tissues other than brain in Alzheimer’s disease. Nature. 1989;341:226–30. 10.1038/341226a0 2528696 10.1038/341226a0

[19] Puig KL, Lutz BM, Urquhart SA, Rebel AA, Zhou X, Manocha GD, et al. Overexpression of mutant amyloid-β protein precursor and presenilin 1 modulates enteric nervous system. J Alzheimers Dis. 2015;44:1263–78. 10.3233/JAD-142259 25408221 10.3233/JAD-142259 PMC6295343

[20] Villemagne VL. Amyloid imaging: past, present and future perspectives. Ageing Res Rev. 2016;30:95–106. 10.1016/j.arr.2016.01.005 26827784 10.1016/j.arr.2016.01.005

[21] Herholz K, Ebmeier K. Clinical amyloid imaging in Alzheimer’s disease. Lancet Neurol. 2011;10:667–. 10.1016/S1474-4422(11)70123-5. 70. 21683932 10.1016/S1474-4422(11)70123-5

[22] Ribaldi F, Chicherio C, Altomare D, Martins M, Tomczyk S, Jelescu I, et al. Brain connectivity and metacognition in persons with subjective cognitive decline (COSCODE): rationale and study design. Alzheimers Res Ther. 2021;13:1–8. 10.1186/s13195-021-00846-z 34034799 10.1186/s13195-021-00846-z PMC8152092

[23] Backhausen LL, Herting MM, Buse J, Roessner V, Smolka MN, Vetter NC. Quality control of structural MRI images applied using FreeSurfer-a hands-on workflow to rate motion artifacts. Front Neurosci. 2016;10:558. 10.3389/fnins.2016.00558 27999528 10.3389/fnins.2016.00558 PMC5138230

[24] Fischl B, FreeSurfer. NeuroImage. 2012;62:774–81. 10.1016/j.neuroimage.2012.01.021 22248573 10.1016/j.neuroimage.2012.01.021 PMC3685476

[25] Klapwijk ET, Kamp F, Van De, Meulen M, Van Der, Peters S, Wierenga LM. Qoala-T: A supervised-learning tool for quality control of freesurfer segmented MRI data. NeuroImage. 2019;189:116–29. 10.1016/j.neuroimage.2019.01.014 30633965 10.1016/j.neuroimage.2019.01.014

[26] Greve DN, Svarer C, Fisher PM, Feng L, Hansen AE, Baare W, et al. Cortical surface-based analysis reduces bias and variance in kinetic modeling of brain PET data. NeuroImage. 2014;92:225–36. 10.1016/j.neuroimage.2013.12.021 24361666 10.1016/j.neuroimage.2013.12.021 PMC4008670

[27] Greve DN, Salat DH, Bowen SL, Izquierdo-Garcia D, Schultz AP, Catana C, et al. Different partial volume correction methods lead to different conclusions: an 18F-FDG-PET study of aging. NeuroImage. 2016;132:334–43. 10.1016/j.neuroimage.2016.02.042 26915497 10.1016/j.neuroimage.2016.02.042 PMC4851886

[28] Jagust WJ, Landau SM, Koeppe RA, Reiman EM, Chen K, Mathis CA, et al. The Alzheimer’s disease neuroimaging initiative 2 PET core: 2015. Alzheimer’s Dement. 2015;11:757–71. 10.1016/j.jalz.2015.05.001 26194311 10.1016/j.jalz.2015.05.001 PMC4510459

[29] Cho SH, Choe YS, Kim YJ, Lee B, Kim HJ, Jang H, et al. Concordance in detecting amyloid positivity between 18F-florbetaben and 18F-flutemetamol amyloid PET using quantitative and qualitative assessments. Sci Rep. 2020;10:19576. 10.1038/s41598-020-76102-5 33177593 10.1038/s41598-020-76102-5 PMC7658982

[30] Rosset A, Spadola L, Ratib O. OsiriX: an open-source software for navigating in multidimensional DICOM images. J Digit Imaging. 2004;17:205–16. 10.1007/s10278-004-1014-6 15534753 10.1007/s10278-004-1014-6 PMC3046608

[31] Hellberg S, Silvola JMU, Liljenbäck H, Kiugel M, Eskola O, Hakovirta H, et al. Amyloid-targeting PET tracer [18F]flutemetamol accumulates in atherosclerotic plaques. Molecules. 2019;24:1072. 10.3390/molecules24061072 30893771 10.3390/molecules24061072 PMC6471324

[32] Wasserthal J, Breit H-C, Meyer MT, Pradella M, Hinck D, Sauter AW, et al. TotalSegmentator: robust segmentation of 104 anatomic structures in CT images. Radiol Artif Intell. 2023;5:e230024. 10.1148/ryai.230024 37795137 10.1148/ryai.230024 PMC10546353

[33] Avants BB, Tustison NJ, Song G, Cook PA, Klein A, Gee JC. A reproducible evaluation of ants similarity metric performance in brain image registration. NeuroImage. 2011;54:2033–44. 10.1016/j.neuroimage.2010.09.025 20851191 10.1016/j.neuroimage.2010.09.025 PMC3065962

[34] Smith SM, Jenkinson M, Woolrich MW, Beckmann CF, Behrens TEJ, Johansen-Berg H, et al. Advances in functional and structural MR image analysis and implementation as FSL. NeuroImage. 2004;23:S208–19. 10.1016/j.neuroimage.2004.07.051 15501092 10.1016/j.neuroimage.2004.07.051

[35] Bolyen E, Rideout JR, Dillon MR, Bokulich NA, Abnet CC, Al-Ghalith GA, et al. Reproducible, interactive, scalable and extensible Microbiome data science using QIIME 2. Nat Biotechnol. 2019;37:852–7. 10.1038/s41587-019-0209-9 31341288 10.1038/s41587-019-0209-9 PMC7015180

[36] Callahan BJ, McMurdie PJ, Rosen MJ, Han AW, Johnson AJA, Holmes SP. DADA2: High-resolution sample inference from illumina amplicon data. Nat Methods. 2016;13:581–3. 10.1038/nmeth.3869 27214047 10.1038/nmeth.3869 PMC4927377

[37] Bokulich NA, Kaehler BD, Rideout JR, Dillon M, Bolyen E, Knight R, et al. Optimizing taxonomic classification of marker-gene amplicon sequences with QIIME 2’s q2-feature-classifier plugin. Microbiome. 2018;6:90. 10.1186/s40168-018-0470-z 29773078 10.1186/s40168-018-0470-z PMC5956843

[38] Kassambara A, rstatix. Pipe-Friendly Framework for Basic Statistical Tests. R package version 0.7.2. https://rpkgs.datanovia.com/rstatix/

[39] Oksanen J, Simpson G, Blanchet FG, Kindt R. vegan: Community Ecology Package. R package version 2.7-0. https://github.com/vegandevs/vegan, https://vegandevs.github.io/vegan/

[40] Kim S. Ppcor: an R package for a fast calculation to Semi-partial correlation coefficients. Commun Stat Appl Methods. 2015;22:665–74. 10.5351/CSAM.2015.22.6.665 26688802 10.5351/CSAM.2015.22.6.665 PMC4681537

[41] Del Tredici K, Braak H. Sporadic Parkinson’s disease: development and distribution of α-synuclein pathology. Neuropathol Appl Neurobiol. 2016;42:33–50. 10.1111/nan.12298 26662475 10.1111/nan.12298

[42] Garretti F, Monahan C, Sloan N, Bergen J, Shahriar S, Kim SW, et al. Interaction of an α-synuclein epitope with HLA-DRB1∗15:01 triggers enteric features in mice reminiscent of prodromal Parkinson’s disease. Neuron. 2023;111:3397–413. 10.1016/j.neuron.2023.07.015 37597517 10.1016/j.neuron.2023.07.015 PMC11068096

[43] Jucker M, Walker LC. Self-propagation of pathogenic protein aggregates in neurodegenerative diseases. Nature. 2013;501:45–51. 10.1038/nature12481 24005412 10.1038/nature12481 PMC3963807

[44] Goedert M. Alzheimer’s and Parkinson’s diseases: the prion concept in relation to assembled Aβ, Tau, and α-synuclein. Science. 2015;349. 10.1126/science.1255555 10.1126/science.1255555 26250687

[45] Visanji NP, Lang AE, Kovacs GG. Beyond the synucleinopathies: alpha synuclein as a driving force in neurodegenerative comorbidities. Transl Neurodegener. 2019;8:1–13. 10.1186/s40035-019-0172-x 31508228 10.1186/s40035-019-0172-x PMC6727368

[46] Schmitt J, Palleis C, Sauerbeck J, Unterrainer M, Harris S, Prix C, et al. Dual-Phase β-Amyloid PET captures neuronal injury and amyloidosis in corticobasal syndrome. Front Aging Neurosci. 2021;13. 10.3389/fnagi.2021.661284 10.3389/fnagi.2021.661284 PMC8155727 34054506

[47] Daneman R, Rescigno M. The gut immune barrier and the Blood-Brain barrier: are they so different?? Immunity. 2009;31:722–35. 10.1016/j.immuni.2009.09.012 19836264 10.1016/j.immuni.2009.09.012

[48] Scalise AA, Kakogiannos N, Zanardi F, Iannelli F, Giannotta M. The blood–brain and gut–vascular barriers: from the perspective of claudins. Tissue Barriers. 2021;9. 10.1080/21688370.2021.1926190 10.1080/21688370.2021.1926190 PMC8489939 34152937

[49] Mestre-Torres J, Lorenzo-Bosquet C, Cuberas-Borrós G, Gironella M, Solans-Laque R, Fernández-Codina A, et al. Utility of the 18 F-Florbetapir positron emission tomography in systemic amyloidosis. Amyloid. 2018;25:109–14. 10.1080/13506129.2018.1467313 29706127 10.1080/13506129.2018.1467313

[50] Akamatsu G, Nishio T, Adachi K, Ikari Y, Senda M. Whole-body biodistribution and the influence of body activity on brain kinetic analysis of the 11 C-PiB PET scan. Radiol Phys Technol. 2017;10:464–74. 10.1007/s12194-017-0419-0 28895034 10.1007/s12194-017-0419-0

[51] Forsythe P, Bienenstock J, Kunze WA. Vagal pathways for microbiome-brain-gut axis communication. Microbial endocrinology: the microbiota-gut-brain axis. Health Disease. 2014;115–33. 10.1007/978-1-4939-0897-4_5 10.1007/978-1-4939-0897-4_5 24997031

[52] Beck G, Hori Y, Hayashi Y, Morii E, Takehara T, Mochizuki H. Detection of phosphorylated Alpha-Synuclein in the muscularis propria of the Gastrointestinal tract is a sensitive predictor for Parkinson’s disease. Parkinsons Dis. 2020;2020. 10.1155/2020/4687530 10.1155/2020/4687530 PMC7530470 33029342

[53] Berge N, Van Den, Ferreira N, Gram H, Mikkelsen TW, Alstrup AKO, Casadei N, et al. Evidence for bidirectional and trans-synaptic parasympathetic and sympathetic propagation of alpha-synuclein in rats. Acta Neuropathol. 2019;138:535–50. 10.1007/s00401-019-02040-w 31254094 10.1007/s00401-019-02040-w PMC6778265

[54] Braak H, Rüb U, Gai WP, Tredici K, Del. Idiopathic Parkinson’s disease: possible routes by which vulnerable neuronal types May be subject to neuroinvasion by an unknown pathogen. J Neural Transm. 2003;110:517–36. 10.1007/s00702-002-0808-2 12721813 10.1007/s00702-002-0808-2

[55] Braak H, Tredici K, Del, Rüb U, Vos RAI, De, Steur ENHJ, Braak E. Staging of brain pathology related to sporadic Parkinson’s disease. Neurobiol Aging. 2003;24:197–211. 10.1016/S0197-4580(02)00065-9 12498954 10.1016/s0197-4580(02)00065-9

[56] Kim S, Kwon S-H, Kam T-I, Panicker N, Karuppagounder SS, Lee S, et al. Transneuronal propagation of pathologic α-synuclein from the gut to the brain models Parkinson’s disease. Neuron. 2019;103:627–41. 10.1016/j.neuron.2019.05.035 31255487 10.1016/j.neuron.2019.05.035 PMC6706297

[57] Ruiz-Riquelme A, Lau HHC, Stuart E, Goczi AN, Wang Z, Schmitt-Ulms G, et al. Prion-like propagation of β-amyloid aggregates in the absence of APP overexpression. Acta Neuropathol Commun. 2018;6. 10.1186/s40478-018-0529-x 10.1186/s40478-018-0529-x PMC5883524 29615128

[58] Sardar Sinha M, Ansell-Schultz A, Civitelli L, Hildesjö C, Larsson M, Lannfelt L, et al. Alzheimer’s disease pathology propagation by exosomes containing toxic amyloid-beta oligomers. Acta Neuropathol. 2018;136. 10.1007/s00401-018-1868-1 10.1007/s00401-018-1868-1 PMC6015111 29934873

[59] Liu G, Yu Q, Zhu H, Tan B, Yu H, Li X, et al. Amyloid-β mediates intestinal dysfunction and enteric neurons loss in Alzheimer’s disease Transgenic mouse. Cell Mol Life Sci. 2023;80:351. 10.1007/s00018-023-04948-9 37930455 10.1007/s00018-023-04948-9 PMC11072809

[60] Zhang L, Wang Y, Xiayu X, Shi C, Chen W, Song N, et al. Altered gut microbiota in a mouse model of Alzheimer’s disease. J Alzheimer’s Disease. 2017;60:1241–57. 10.3233/JAD-170020 29036812 10.3233/JAD-170020

[61] Pellegrini C, Daniele S, Antonioli L, Benvenuti L, D’antongiovanni V, Piccarducci R, et al. Prodromal intestinal events in Alzheimer’s disease (AD): colonic dysmotility and inflammation are associated with enteric ad-related protein deposition. Int J Mol Sci. 2020;21:3523. 10.3390/ijms21103523 32429301 10.3390/ijms21103523 PMC7278916

[62] Eisele YS, Obermüller U, Heilbronner G, Baumann F, Kaeser SA, Wolburg H, et al. Peripherally applied Aβ-containing inoculates induce cerebral β-amyloidosis. Science. 2010;330:980–2. 10.1126/science.1194516 20966215 10.1126/science.1194516 PMC3233904

[63] Sun Y, Sommerville NR, Liu JYH, Ngan MP, Poon D, Ponomarev ED, et al. Intra-gastrointestinal amyloid-β1–42 oligomers perturb enteric function and induce Alzheimer’s disease pathology. J Physiol. 2020;598:4209–23. 10.1113/jp279919 32617993 10.1113/JP279919 PMC7586845

[64] Chen C, Zhou Y, Wang H, Alam A, Kang SS, Ahn EH, et al. Gut inflammation triggers C/EBPβ/δ-secretase‐dependent gut‐to‐brain propagation of Aβ and Tau fibrils in Alzheimer’s disease. EMBO J. 2021;40:e106320. 10.15252/embj.2020106320 34260075 10.15252/embj.2020106320 PMC8408610

[65] Chen C, Liao J, Xia Y, Liu X, Jones R, Haran J, et al. Gut microbiota regulate Alzheimer’s disease pathologies and cognitive disorders via PUFA-associated neuroinflammation. Gut. 2022;71:2233–52. 10.1136/gutjnl-2021-326269 35017199 10.1136/gutjnl-2021-326269 PMC10720732

[66] Marizzoni M, Mirabelli P, Mombelli E, Coppola L, Festari C, Lopizzo N, et al. A peripheral signature of Alzheimer’s disease featuring microbiota-gut-brain axis markers. Alzheimers Res Ther. 2023;15:101. 10.1186/s13195-023-01218-5 37254223 10.1186/s13195-023-01218-5 PMC10230724

[67] Engels C, Ruscheweyh HJ, Beerenwinkel N, Lacroix C, Schwab C. The common gut microbe Eubacterium hallii also contributes to intestinal propionate formation. Front Microbiol. 2016;7:713. 10.3389/fmicb.2016.00713 27242734 10.3389/fmicb.2016.00713 PMC4871866

[68] Louis P, Young P, Holtrop G, Flint HJ. Diversity of human colonic butyrate-producing bacteria revealed by analysis of the butyryl-CoA:acetate CoA-transferase gene. Environ Microbiol. 2010;12:304–14. 10.1111/j.1462-2920.2009.02066.x 19807780 10.1111/j.1462-2920.2009.02066.x

[69] Stadlbauer V, Engertsberger L, Komarova I, Feldbacher N, Leber B, Pichler G, et al. Dysbiosis, gut barrier dysfunction and inflammation in dementia: A pilot study. BMC Geriatr. 2020;20:248. 10.1186/s12877-020-01644-2 32690030 10.1186/s12877-020-01644-2 PMC7372911

[70] Nishiwaki H, Ito M, Ishida T, Hamaguchi T, Maeda T, Kashihara K, et al. Meta-Analysis of gut dysbiosis in Parkinson’s disease. Mov Disord. 2020;35. 10.1002/mds.28119 10.1002/mds.28119 32557853

[71] Sanguinetti E, Collado MC, Marrachelli VG, Monleon D, Selma-Royo M, Pardo-Tendero MM, et al. Microbiome-metabolome signatures in mice genetically prone to develop dementia, fed a normal or fatty diet. Sci Rep. 2018;8. 10.1038/s41598-018-23261-1 10.1038/s41598-018-23261-1 PMC5861049 29559675

[72] Kageyama A, Benno Y. Coprobacillus catenaformis gen. Nov., Sp. Nov., a new genus and species isolated from human feces. Microbiol Immunol. 2000;44:23–8. 10.1111/j.1348-0421.2000.tb01242.x 10711596 10.1111/j.1348-0421.2000.tb01242.x

[73] Ye J, Lv L, Wu W, Li Y, Shi D, Fang D, et al. Butyrate protects mice against Methionine–Choline-Deficient Diet-Induced Non-alcoholic steatohepatitis by improving gut barrier function, attenuating inflammation and reducing endotoxin levels. Front Microbiol. 2018;9. 10.3389/fmicb.2018.01967 10.3389/fmicb.2018.01967 PMC6111843 30186272

[74] Stein RR, Bucci V, Toussaint NC, Buffie CG, Rätsch G, Pamer EG, et al. Ecological modeling from Time-Series inference: insight into dynamics and stability of intestinal microbiota. PLoS Comput Biol. 2013;9. 10.1371/journal.pcbi.1003388 10.1371/journal.pcbi.1003388 PMC3861043 24348232

[75] Aasmets O, Lüll K, Lang JM, Pan C, Kuusisto J, Fischer K, et al. Machine learning reveals Time-Varying microbial predictors with complex effects on glucose regulation. mSystems. 2021;6. 10.1128/msystems.01191-20 10.1128/mSystems.01191-20 PMC8573957 33594006

[76] Yu J, Lee KN, Kim HS, Han K, Lee SH. Cumulative effect of impaired fasting glucose on the risk of dementia in middle-aged and elderly people: a nationwide cohort study. Sci Rep. 2023;13. 10.1038/s41598-023-47566-y 10.1038/s41598-023-47566-y PMC10667225 37996487

[77] Perov S, Lidor O, Salinas N, Golan N, Tayeb-Fligelman E, Deshmukh M, et al. Structural insights into curli CsgA cross-β fibril architecture inspire repurposing of anti-amyloid compounds as anti-biofilm agents. PLoS Pathog. 2019;15. 10.1371/journal.ppat.1007978 10.1371/journal.ppat.1007978 PMC6748439 31469892

[78] Chen SG, Stribinskis V, Rane MJ, Demuth DR, Gozal E, Roberts AM, et al. Exposure to the functional bacterial amyloid protein curli enhances alpha-synuclein aggregation in aged Fischer 344 rats and Caenorhabditis elegans. Sci Rep. 2016;6:34477. 10.1038/srep34477 27708338 10.1038/srep34477 PMC5052651

[79] Friedland RP, Chapman MR. The role of microbial amyloid in neurodegeneration. PLoS Pathog. 2017. 10.1371/journal.ppat.1006654. 13. 29267402 10.1371/journal.ppat.1006654 PMC5739464

[80] Moir RD, Lathe R, Tanzi RE. The antimicrobial protection hypothesis of Alzheimer’s disease. Alzheimer’s Dement. 2018;14:1602–14. 10.1016/j.jalz.2018.06.3040 30314800 10.1016/j.jalz.2018.06.3040

[81] Pellegrini C, Fornai M, D’Antongiovanni V, Antonioli L, Bernardini N, Derkinderen P. The intestinal barrier in disorders of the central nervous system. Lancet Gastroenterol Hepatol. 2023;8:66–80. 10.1016/s2468-1253(22)00241-2 36334596 10.1016/S2468-1253(22)00241-2

[82] Pedicord VA, Mucida D. A sledgehammer breaks glass but Forges steel: BacteriA adhesion shapes gut immunity. Cell. 2015;16:273–4. 10.1016/j.cell.2015.09.040 10.1016/j.cell.2015.09.040 26451476

[83] Bernardazzi C, Xu H, Tong H, Laubitz D, da Paz VF, Curiel L, et al. An indisputable role of NHE8 in mucosal protection. Am J Physiol Gastrointest Liver Physiol. 2020;319:421–31. 10.1152/ajpgi.00246.2020 10.1152/ajpgi.00246.2020 PMC7654648 32755385

[84] Katsumata Y, Fardo DW, Bachstetter AD, Artiushin SC, Wang WX, Wei A, et al. Alzheimer disease pathology-associated polymorphism in a complex variable number of tandem repeat region within the MUC6 gene, near the AP2A2 gene. J Neuropathol Exp Neurol. 2020;79:3–21. 10.1093/jnen/nlz116 31748784 10.1093/jnen/nlz116 PMC8204704

[85] Senda M, Brooks DJ, Farrar G, Somer EJ, Paterson CL, Sasaki M, et al. The clinical safety, biodistribution and internal radiation dosimetry of flutemetamol (18F) injection in healthy Japanese adult volunteers. Ann Nucl Med. 2015;29:627–35. 10.1007/s12149-015-0986-2 26044876 10.1007/s12149-015-0986-2 PMC4526582

[86] Odamaki T, Kato K, Sugahara H, Hashikura N, Takahashi S, Xiao JZ, et al. Age-related changes in gut microbiota composition from newborn to centenarian: A cross-sectional study. BMC Microbiol. 2016;16:1–12. 10.1186/s12866-016-0708-5 27220822 10.1186/s12866-016-0708-5 PMC4879732

[87] Lowe VJ, Lundt E, Knopman D, Senjem ML, Gunter JL, Schwarz CG, et al. Comparison of [18F]Flutemetamol and [11 C]Pittsburgh Compound-B in cognitively normal young, cognitively normal elderly, and Alzheimer’s disease dementia individuals. Neuroimage Clin. 2017;16:295–302. 10.1016/j.nicl.2017.08.011 28856092 10.1016/j.nicl.2017.08.011 PMC5565786

[88] Drzezga A, Grimmer T, Henriksen G, Mühlau M, Perneczky R, Miederer I, et al. Effect of APOE genotype on amyloid plaque load and Gray matter volume in alzheimer disease. Neurology. 2009;72:1487–94. 10.1212/wnl.0b013e3181a2e8d0 19339712 10.1212/WNL.0b013e3181a2e8d0

[89] Tran TTT, Corsini S, Kellingray L, Hegarty C, Le Gall G, Narbad A, et al. APOE genotype influences the gut Microbiome structure and function in humans and mice: relevance for Alzheimer’s disease pathophysiology. FASEB J. 2019;33:8221–31. 10.1096/fj.201900071r 30958695 10.1096/fj.201900071R PMC6593891

