# Supplementary material for: [18F]flutemetamol uptake in the colon of a memory clinic population and its association with brain amyloidosis and the gut microbiota profile: an exploratory study
Source: Eur J Nucl Med Mol Imaging. 2025 May 2;52(12):4591–603. doi: 10.1007/s00259-025-07299-8 (PMC12491377; doi:10.1007/s00259-025-07299-8)

**[18F]flutemetamol uptake in the colon of a memory clinic population and its association with brain amyloidosis and the gut microbiota profile: an exploratory study.**

Giulia Quattrini^1^, Elena Gatti^1^, Débora Elisa Peretti^2^, Marco Aiello^3^, Claire Chevalier^4,5^, Aurelien Lathuiliere^4,5^, Rahel Park^4,5^, Michela Pievani^1^, Marco Salvatore^3^, Max Scheffler^6^, Annamaria Cattaneo^7,8^, Giovanni B Frisoni^4,5^, Valentina Garibotto^2,9,10^, Moira Marizzoni^6^*

**Supplementary information**

**Supplementary Figure 1**. Maximum intensity projection images of the abdominopelvic body region on [18F]flutemetamol (FMM) PET/CT scans for both early (e-FMM) and late (l-FMM) phases from an amyloid negative (panel A) and an amyloid positive (panel B) participant.


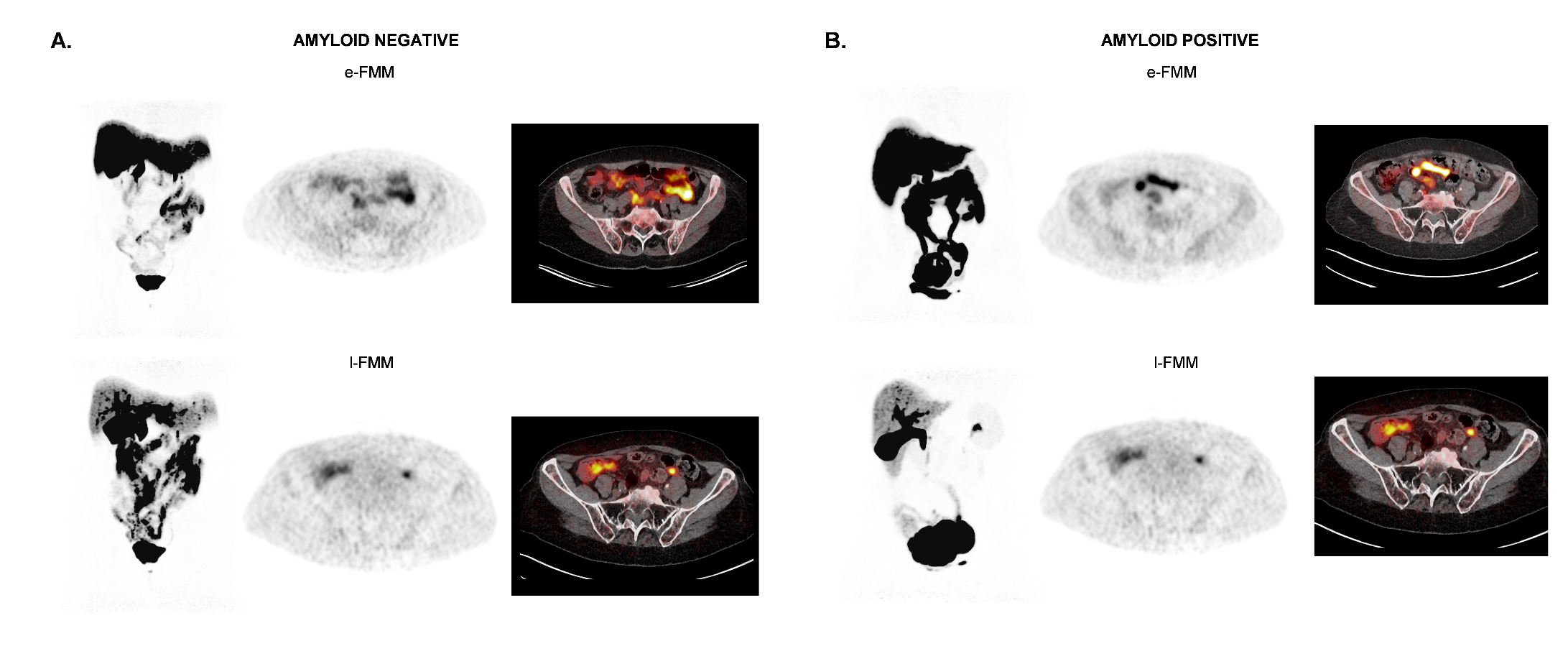


**Supplementary Figure 2.** APOE ε4 carrier status influences the mean SUVr uptake in the sigmoid colon in the late phase when using manual tracing for measurements (panel A). It does not influence maximum SUVr or measurements obtained by automated segmentation (panel B).


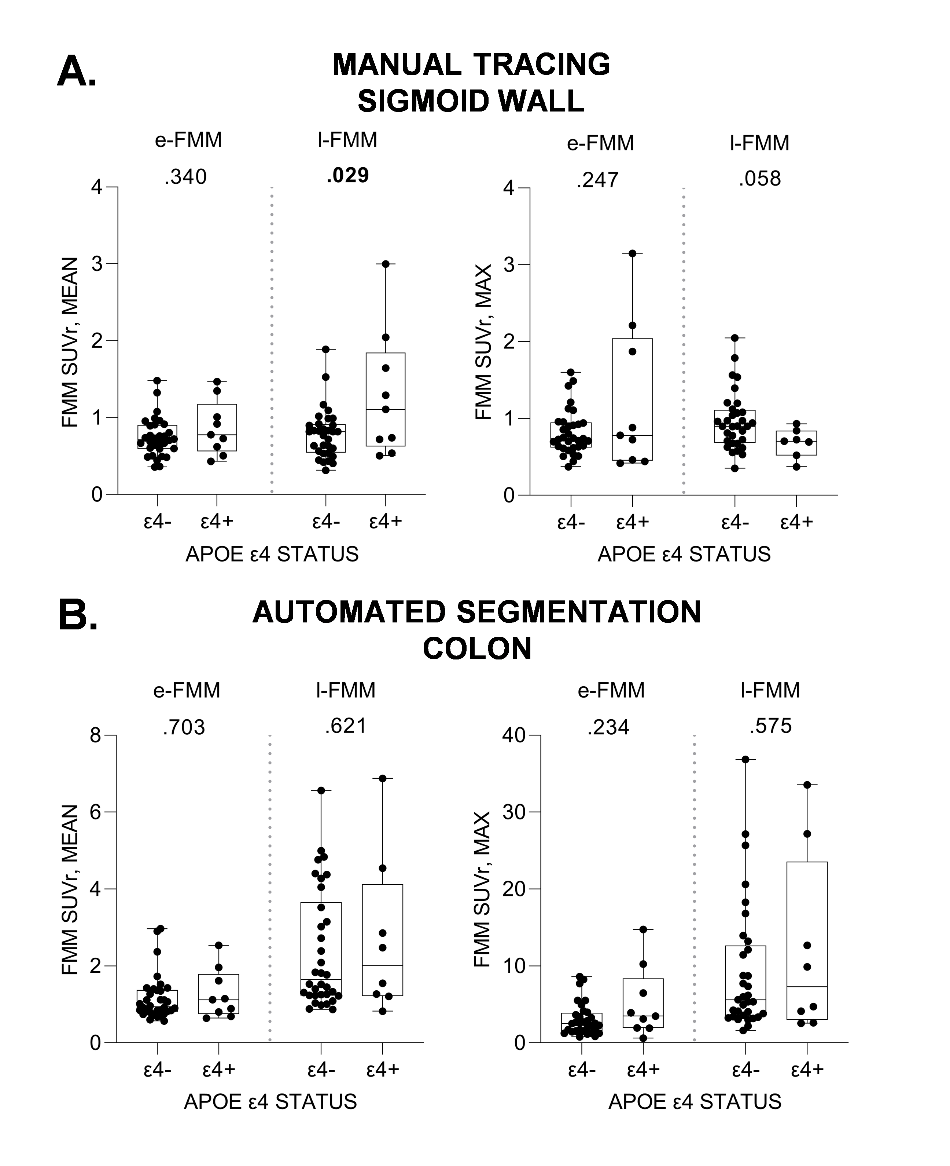


**Supplementary Figure 3.** Amyloid pathology status in the brain (negative [A-] / positive [A+]) did not influence alpha diversity measured by the Shannon index (panel A), and beta diversity measured by Bray Curtis and Jaccard distances (panel B and C, respectively). Beta diversity metrics were computed using normalized data.


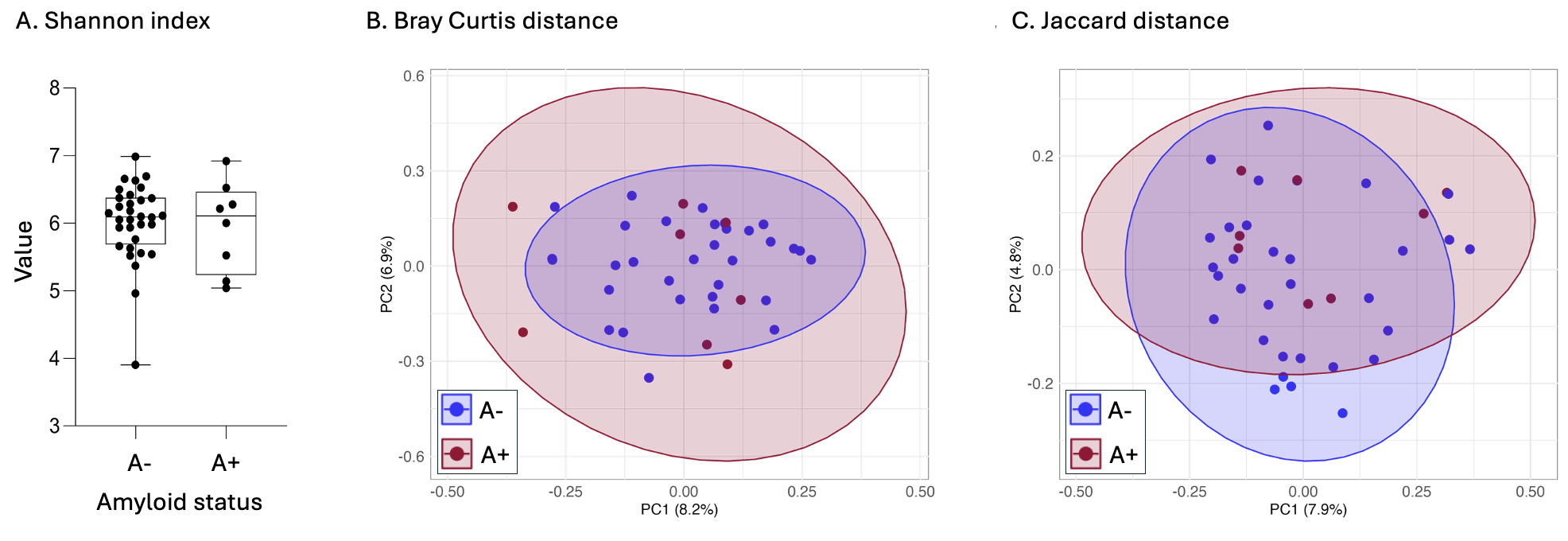


**Supplementary Figure 4.** APOE ε4 carrier status does not influence alpha (panel A) and beta (panel B) diversity measures, but it does affect fecal microbiota composition at the genus level (panel C, linear discriminant analysis (LDA) effect size (LEfSe) algorithm, LDA >3). Beta diversity metrics were computed using normalized data.


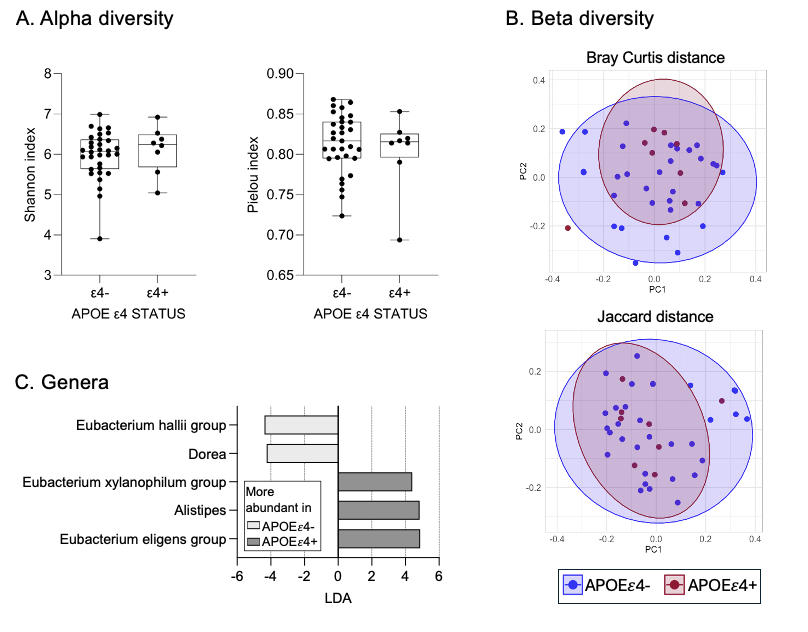

Supplement: Supplementary file 1 — Supplementary Material 1 [file 259_2025_7299_MOESM1_ESM.docx]
